# Impact of a Continuous Quality Improvement Committee on Outcomes for Patients Undergoing Percutaneous Coronary Intervention: A Single-Center Retrospective Cohort Study

**DOI:** 10.1016/j.jscai.2026.105577

**Published:** 2026-08-20

**Authors:** Arnav Kumar Singla, Basmah Fayaz, Christopher Heinrich, Samuel Miller, John Simpson, Samantha Mutai, Gilgamish Maloul, Malek Lababidi, Julie Ahrenholz, Weili Zheng, Faisal Hasan, Hussam S. Suradi

**Affiliations:** a Rush University Medical Center, Chicago, Illinois; b Rush Medical College, Chicago, Illinois; c UIC Health, Chicago, Illinois

**Keywords:** bleeding avoidance, nephroprotection, patient safety, quality improvement, radial access, same-day discharge

## Abstract

**Background:**

Continuous quality improvement (CQI) programs aim to enhance procedural safety and efficiency in percutaneous coronary intervention (PCI). This analysis evaluates changes in PCI practice patterns and outcomes after implementation of a multidisciplinary catheterization laboratory CQI committee at a large academic center.

**Methods:**

A retrospective cohort study was conducted regarding PCI procedures from January 2018 to June 2024 using National Cardiovascular Data Registry CathPCI data. Outcomes and practice patterns were compared before (pre-CQI: 2018-October 2022) versus after CQI implementation (post-CQI: November 2022-2024). Prespecified primary end points were post-PCI acute kidney injury, periprocedural hemoglobin change, and same-day discharge.

**Results:**

Among 2759 PCI cases (1783 pre-CQI; 976 post-CQI), monthly PCI volume increased (30.7-48.2 cases/mo, *P* < .001). Post-CQI, radial access use increased (33.4%-58.9%, *P* < .001) and mean contrast volume decreased (188-153 mL, *P* < .001). Primary outcomes improved post-CQI: acute kidney injury decreased (14.3%-9.7%, *P* = .01), mean periprocedural hemoglobin change decreased (0.65-0.54 g/dL, *P* = .004), and same-day discharge increased (13.0%-24.7%, *P* < .001). Overall complication rates and in-hospital mortality remained stable.

**Conclusions:**

Implementation of a catheterization laboratory CQI committee was temporally associated with improved PCI safety and discharge efficiency alongside increased procedural volume and evolving case mix. These findings support CQI-driven approaches to optimize PCI care delivery.

## Introduction

Percutaneous coronary intervention (PCI) has transformed over the past decades, driven by advances in device technology, pharmacotherapy, intravascular imaging, and mechanical circulatory support (MCS).^1^^,^^2^ These developments have expanded the range of coronary lesion complexity and patient populations treatable with PCI.^3^^,^^4^ As contemporary PCI is increasingly performed in older patients with greater comorbidities and procedural risk, systematic quality oversight is needed to ensure that technical advances translate into safe, consistent, and patient-centered care.^5^

Continuous quality improvement (CQI) in health care seeks to identify opportunities for improvement through systematic performance evaluation, case review, staff education, and use of nationally standardized benchmarks. In PCI, CQI initiatives have been associated with improved adherence to evidence-based practices, increased use of bleeding avoidance strategies, and reductions in selected procedural complications.^6^, ^7^, ^8^, ^9^, ^10^, ^11^ National registries such as the National Cardiovascular Data Registry (NCDR) provide benchmarks that can support local quality improvement efforts.^12^^,^^13^

Despite broader adoption of catheterization lab CQI programs, limited evidence describes how CQI program features influence practice patterns and patient-centered outcomes. The association between implementation of a multidisciplinary catheterization lab CQI program and changes in PCI safety and efficiency outcomes at a large urban academic medical center is evaluated here. Prespecified outcomes included acute kidney injury (AKI), periprocedural hemoglobin change, and same-day discharge (SDD); procedural volume was reported as a contextual marker of institutional throughput and evolving case mix rather than as a direct quality metric.

## Materials and methods

### Study design and data sources

This retrospective observational cohort study included adult patients undergoing PCI at a single institution from January 2018 to June 2024. Patient data were obtained from electronic medical records and the NCDR CathPCI Registry, including demographic characteristics, clinical characteristics, procedural details, hospital metrics, periprocedural complications, mortality indicators, and AKI-related risk factors and outcomes. The study was reviewed by an Institutional Review Board and deemed exempt as quality improvement and operational reporting. Clinical, procedural, and in-hospital outcome variables were extracted from the NCDR CathPCI Registry and analyzed according to registry definitions. Variables derived for this analysis included CQI period assignment based on procedure date relative to committee implementation, monthly PCI volume calculated from the number of PCI procedures performed per calendar month, and SDD defined as discharge on the same calendar date as PCI.

Procedure completion before vs after 15:00 was used for exploratory SDD analyses because cases completed after 15:00 generally required overnight observation due to limited postcatheterization recovery unit availability rather than clinical ineligibility alone.

### CQI committee

In October 2022, a multidisciplinary Cardiac Catheterization Laboratory Quality Committee was established and led by the Cardiac Catheterization Laboratory Director. The committee reviewed NCDR CathPCI quality metrics, internal performance data, complications and outlier cases, and proposed protocol updates. Membership included interventional cardiologists, nursing leadership, fellows, and catheterization laboratory technologists. The committee met monthly, and protocol changes were approved by consensus and disseminated through staff in-services, preprocedure time-outs, and postprocedure huddles.

### End points and definitions

Committee-led interventions targeted bleeding avoidance, AKI mitigation, and discharge efficiency. Based on these aims the prespecified primary end points were post-PCI AKI, periprocedural change in hemoglobin, and SDD. These outcomes were selected because of their established association with procedural safety, patient recovery, and institutional performance in PCI.^14^, ^15^, ^16^, ^17^ Secondary and exploratory outcomes included NCDR-observed bleeding, transfusion, in-hospital mortality, length of stay, radial access use, contrast volume, and staged PCI. Frailty was assessed using registry-defined Rockwood Clinical Frailty Scale categories, with severe frailty indicating need for assistance with basic activities of daily living or greater dependency.^18^

### Bleeding avoidance and access protocols

To decrease postprocedural bleeding rates, the committee implemented a standardized “radial-first” access strategy for patients, with standard exceptions limited to cases requiring large-bore access or MCS. Universal ultrasound guidance was standardized for all vascular access (both radial and femoral). If femoral access was obtained, routine limited iliofemoral angiography was employed for closure evaluation and device closure (Angio-Seal [Terumo]/Perclose [Abbott]) were used whenever possible. Furthermore, standardized postprocedure monitoring protocols for both radial and femoral access sites were embedded into electronic health record order sets and nursing workflows to standardize assessment parameters and timeframes for achieving hemostasis. For radial access, depending on the amount of heparin used, the transradial band protocol changes from 1 to 2 hours before air is released from the band. In particularly complex cases, this time can be extended. For femoral access, lay flat time is increased based on amount of heparin used, complexity of the access, and type of closure (manual vs device).

### AKI mitigation and contrast stewardship

To reduce AKI risk, the committee introduced comprehensive contrast stewardship and periprocedural hydration protocols. Preprocedural intravenous fluid administration criteria were standardized using an estimated glomerular filtration rate (eGFR)-guided approach embedded directly into the preprocedural order set used for each PCI case. No eGFR cutoff was utilized preprocedurally. Intraprocedurally, left ventricular end-diastolic pressures were routinely obtained if a patient had eGFR <60 mL/min/1.73 m² and/or any baseline renal dysfunction to guide postprocedural hydration order sets; appropriate adjustments/exceptions were made for patients with contraindications to aggressive volume administration including decompensated heart failure, shock, or acute valve syndromes. Contrast limit was determined by the equation 3.7 × eGFR.^19^ Additionally, the committee emphasized contrast volume minimization strategies, strongly encouraging operators to perform a structured “contrast pause” once ∼50% of the recommended contrast volume threshold had been used. Operators were further encouraged to stage multivessel PCI procedures whenever there was concern for exceeding safe contrast thresholds or precipitating AKI (Appendix A).

### SDD pathway

The SDD pathway included preprocedural patient education, nursing-driven eligibility criteria, and standardized postprocedure monitoring. Planned overnight admission was reserved for high-risk interventions, late-day procedures, transportation barriers, or periprocedural complications. SDD criteria included access site hemostasis, stable hemodynamics, ambulation, absence of complications, reliable transportation, and follow-up.

For further details regarding methods and protocols used to mitigate bleeding, renal injury, in which improving overall outcomes and quality metrics with SDD, please see Appendix A.

### Statistical analysis

All data, once sourced from the NCDR, was imported into Microsoft Excel (2026) and the Real Statistics Resource Pack (Release 9.8; 2026) for statistical analyses and comparisons. Variables are summarized as mean ± SD, median with IQR, and categorical variables with sample size (n) with associated percentage of total. Baseline, procedural, and clinical characteristics/outcomes were compared between pre-/post-CQI periods using 2-sample *t* tests or Wilcoxon rank-sum tests for continuous variables and chi-square or Fisher exact tests for categorical variables, as appropriate.

Based on the CQI committee’s targeted aims, 3 primary end points were prespecified: post-PCI AKI, periprocedural change in hemoglobin (Hgb drop) as a bleeding safety signal, and SDD. To control the family-wise type I error rate across these primary end points, Holm-Bonferroni adjustment was applied (two-sided overall *α* = .05). Under this approach, ordered primary end point *P* values were compared sequentially with thresholds of .0167, .025, and .05, respectively. Holm-Bonferroni adjusted *P* values are reported for the 3 primary end points. All other comparisons (including baseline characteristics, procedural/process measures, and additional clinical outcomes such as NCDR-defined observed bleeding and transfusion) were considered secondary or exploratory and are reported with nominal *P* values.

Temporal changes in monthly PCI volume, radial access use, and SDD rates were evaluated using segmented regression and interrupted time-series analyses. Models included terms for time, intervention period, and postintervention time trend to estimate pre-CQI slope, immediate level change after CQI implementation, and post-CQI slope. Monthly outcomes were modeled as counts or proportions, as appropriate.

For revised analyses addressing missing outcome data, AKI analyses were restricted to patients with evaluable baseline and postprocedure creatinine after exclusion of patients discharged the same-day and patients receiving chronic hemodialysis. Baseline characteristics of the AKI-evaluable cohort were compared between pre-/post-CQI periods. Multivariable logistic regression was used to assess the association between post-CQI status and AKI, adjusting for age, heart failure, baseline eGFR, cardiogenic shock, and MCS use. A sensitivity model additionally included radial access and contrast volume. Complete-case analysis was used, and no missing values were imputed. Hemoglobin-drop analyses included all PCI cases because baseline and postprocedure hemoglobin values were available for all patients, including those discharged the same-day. Therefore, no patients were excluded from the hemoglobin-drop analysis due to same-day discharge status or missing hemoglobin data.

## Results

### Patient characteristics

Between 2018 and mid-2024, a total of 2759 PCI procedures were performed at this institution (1783 pre-CQI and 976 post-CQI). The mean patient age post-CQI was slightly higher (67.3 vs 65.4 years, *P* < .001), and the proportion of patients identifying as Hispanic/Latino increased (17.9% vs 16.4%, *P* < .001). Other characteristics such as sex, race, and body mass index did not differ between periods (Table 1).

**Table 1:** Patient and case characteristics

Characteristics	Total	Pre-CQI (2018 - Oct 2022)	Post-CQI (Nov 2022 - 2024)	*P* value
Total PCIs	2759	1783	976	
PCIs per month	35.4	30.7	48.2	<.001
Age, y	66.0 ± 12.0	65.4 ± 11.9	67.3 ± 12.0	<.001
Race/ethnicity	2722	1758	964	.66
White	1638 (60.2)	1051 (59.8)	587 (60.9)	
Black	840 (30.9)	551 (31.3)	289 (30.0)	
Asian	138 (5.1)	85 (4.8)	53 (5.5)	
Hispanic/Latino	65 (2.4)	41 (2.3)	24 (2.5)	
Other	41 (1.5)	30 (1.7)	11 (1.1)	
Sex				.63
Male	1821 (66.0)	1183 (66.3)	638 (65.4)	
Female	938 (34.0)	600 (33.7)	338 (34.6)	
Body mass index, kg/m^2^	29.9 ± 6.58	30.0 ± 6.72	29.6 ± 6.31	.28
Medical history				
Hypertension^a^	1151 (85.6)	325 (88.3)	826 (84.6)	.08
Diabetes mellitus	1326 (48.1)	844 (47.3)	482 (49.4)	.29
Heart failure	774 (28.0)	424 (23.7)	350 (35.9)	<.001
Anemia	386 (14.0)	228 (12.8)	158 (16.2)	.02
Prior PCI	1060 (38.4)	691 (38.8)	369 (37.8)	.64
CVA	462 (16.7)	300 (16.8)	162 (16.6)	.89
PAD	314 (11.4)	206 (11.6)	108 (11.1)	.71
Lung disease	281 (10.2)	185 (10.4)	96 (9.8)	.66
Severe frailty	168 (6.09)	36 (2.02)	132 (13.5)	.048
Renal function				
eGFR 45-60 mL/min/1.73 m²	353 (12.8)	231 (13.0)	122 (12.5)	.74
eGFR 30-44 mL/min/1.73 m²	198 (7.2)	114 (6.4)	84 (8.6)	.04
Hemodialysis or eGFR <30 mL/min/1.73 m²	475 (17.2)	293 (16.4)	182 (18.6)	.14
eGFR, mL/min/1.73 m²	64.3 ± 31.5	66.6 ± 31.4	62.4 ± 31.6	<.001
Ejection fraction, %	50.6 ± 13.2	51.2 ± 12.3	49.3 ± 15.0	.003
CV instability^b^				<.001
Elective	1280 (46.4)	820 (46.0)	460 (47.2)	
Urgent	1039 (37.7)	672 (37.7)	367 (37.6)	
Emergency	313 (11.4)	233 (13.1)	80 (8.2)	
Shock without salvage	112 (4.1)	50 (2.8)	63 (6.0)	
Salvage	13 (0.5)	7 (0.4)	6 (0.6)	
STEMI	318 (11.5)	212 (11.9)	106 (10.9)	.42
NSTEMI	908 (32.9)	623 (34.9)	285 (29.2)	<.001
MCS	156 (5.7)	99 (5.6)	57 (5.8)	.69
Inserted prior to PCI	58 (2.1)	20 (1.1)	38 (3.9)	<.001
Cardiogenic shock	81 (2.9)	44 (2.5)	37 (3.8)	.06
Cardiac arrest	53 (1.9)	30 (1.7)	23 (2.4)	.24

Values are mean ± SD or n (%). CQI, continuous improvement; CV, cardiovascular; CVA, cerebrovascular accident; DM, diabetes mellitus; eGFR, estimated glomerular filtration rate; LM, left main artery; MCS, mechanical circulatory support; NSTEMI, non–ST-elevation myocardial infarction; PAD, peripheral arterial disease; PCI, percutaneous coronary intervention; pLAD, proximal left anterior descending artery; STEMI, ST-elevation myocardial infarction.

a 1344 patients out of sample had documented hypertension, 386 patients pre-CQI and 976 patients post-CQI. Percentages reflect hypertension among those with documented “yes” and “no” responses.

b More emergency cases in pre-CQI and shock without salvage cases in post-CQI.

Despite similar overall comorbidity burden by traditional risk scores, several higher-risk features were present post-CQI, including heart failure (35.9% vs 23.7%; *P* < .001), anemia (16.2% vs 12.8%; *P* = .02), and severe frailty (13.5% vs 2.0%; *P* = .048) (Table 1). Cardiogenic shock and other acute instability were modestly more frequent post-CQI, whereas NSTEMI/acute coronary syndrome were less common (29.2% vs 34.9%; *P* < .001). The use of pre-PCI MCS increased from 1.1% to 3.9% (*P* < .001), consistent with greater acuity and procedural complexity.

### AKI

Renal outcomes demonstrated favorable changes in the post-CQI period. The mean IV contrast used per case decreased from 188 mL pre-CQI to 153 mL post-CQI (*P* < .001), reflecting adherence to contrast sparing protocols. AKI incidence post-PCI declined from 14.3% to 9.7% (*P* = .01), corresponding to these nephroprotective efforts (Table 2). Of 2759 PCI cases, 1110 were excluded from AKI analysis due to SDD (*n* = 312; pre-CQI *n* = 60, post-CQI *n* = 252), chronic hemodialysis (*n* = 112; pre-CQI *n* = 34, post-CQI *n* = 78), or missing baseline and/or postprocedure Cr data after these exclusions (*n* = 686; pre-CQI *n* = 597, post-CQI *n* = 89). The final AKI-evaluable cohort included 1649 cases, including 1092 pre-CQI and 557 post-CQI cases (Figure 1; Appendix B). The Holm-Bonferroni-adjusted *P* value for reduction in AKI was .01. In a primary multivariable model adjusting for age, heart failure, baseline eGFR, cardiogenic shock, and MCS use, the post-CQI period remained associated with lower odds of AKI (adjusted OR, 0.63; 95% CI, 0.45-0.87; *P* = .006). This finding was consistent in a sensitivity model additionally adjusting for radial access and contrast volume (adjusted OR, 0.47; 95% CI, 0.33-0.67; *P* < .001) (Appendix B, Appendix C).

**Table 2:** Case and postprocedural complications.

Characteristics	Total	Pre-CQI (2018 - Oct 2022)	Post-CQI (Nov 2022 - 2024)	*P* value
Access				<.001
Radial	1170 (42.4)	595 (33.4)	575 (58.9)	
Femoral	1589 (57.6)	1187 (66.6)	401 (41.1)	
Predicted mortality, %	1.6 ± 6.2	1.5 ± 5.8	2.0 ± 7.5	.23
Deceased patients	47 (1.7)	27 (1.5)	20 (2.0)	.32
Etiology of death				.11
MI	21 (43.8)	10 (35.7)	11 (55.0)	
SCD	8 (16.7)	8 (28.6)	0 (0)	
Other	5 (10.4)	4 (14.3)	1 (5.0)	
HF	4 (8.3)	2 (7.1)	2 (10.0)	
Pulmonary	4 (8.3)	1 (3.6)	3 (15.0)	
Infection	4 (8.3)	2 (7.1)	2 (10.0)	
Stroke	1 (2.1)	0 (0)	1 (5.0)	
Hemorrhage	1 (2.1)	1 (3.6)	0 (0)	
Staged PCI	206 (7.6)	56 (3.1)	150 (16.5)	<.01
Chronic total occlusion	170 (6.2)	105 (5.9)	65 (6.7)	.43
In-stent thrombosis	30 (1.1)	25 (1.4)	5 (0.5)	.01
Observed bleed	68 (2.5)	47 (2.6)	21 (2.2)	.38
Predicted % bleed, %	3.6 ± 5.8	3.7 ± 6.0	3.4 ± 5.6	.24
Pre-cath Hgb	12.5 ± 2.3	12.7 ± 2.4	12.2 ± 2.1	<.001
Hgb drop	0.61 ± 1.0	0.65 ± 1.1	0.54 ± 0.9	.004 (adj .008)^b^
Bleeding site				
Access	10 (0.4)	7 (0.4)	3 (0.3)	.71
Gastrointestinal	10 (0.4)	7 (0.4)	3 (0.3)	.71
Genitourinary	2 (0.1)	2 (0.1)	0 (0)	.15
Hematoma	14 (0.5)	11 (0.6)	3 (0.3)	.22
Other	9 (0.3)	5 (0.3)	4 (0.4)	.59
Retroperitoneal	4 (0.1)	4 (0.2)	0 (0)	.045
Blood transfusion given	79 (2.9)	40 (2.2)	39 (4.0)	.01
Tamponade	3 (0.1)	3 (0.2)	0 (0)	.08
Hemorrhagic CVA	0 (0)	0 (0)	0 (0)	
Observed AKI^a^	210 (12.7)	156 (14.3)	54 (9.7)	.01 (adj .01)^b^
Observed hemodialysis	12 (0.4)	10 (0.6)	2 (0.2)	.18
Pre-cath Cr	2.0 ± 2.5	1.9 ± 2.3	1.3 ± 1.1	<.001
Post-cath Cr	1.6 ± 2.6	2.0 ± 2.5	1.4 ± 1.2	<.001
Contrast, mL	175 ± 77.2	188 ± 77.6	153 ± 78.8	<.001

Values are n (%) or mean ± SD. AKI, acute kidney injury; CQI, continuous quality improvement; Cr, creatinine; CVA, cerebrovascular accident; Hgb, hemoglobin; HF, heart failure; PCI, percutaneous coronary intervention; SCD, sudden cardiac death.

a See Figure 2 for explanation of missing data; only 1649/2759 (60%) of patients in this cohort had available AKI data.

b Adjusted *P* values are Holm-Bonferroni corrected across the 3 prespecified primary end points (AKI, mean Hgb drop, and SDD). Adjusted *P* values are shown only for primary end points; all other *P* values are nominal/exploratory.

**Figure 1:**
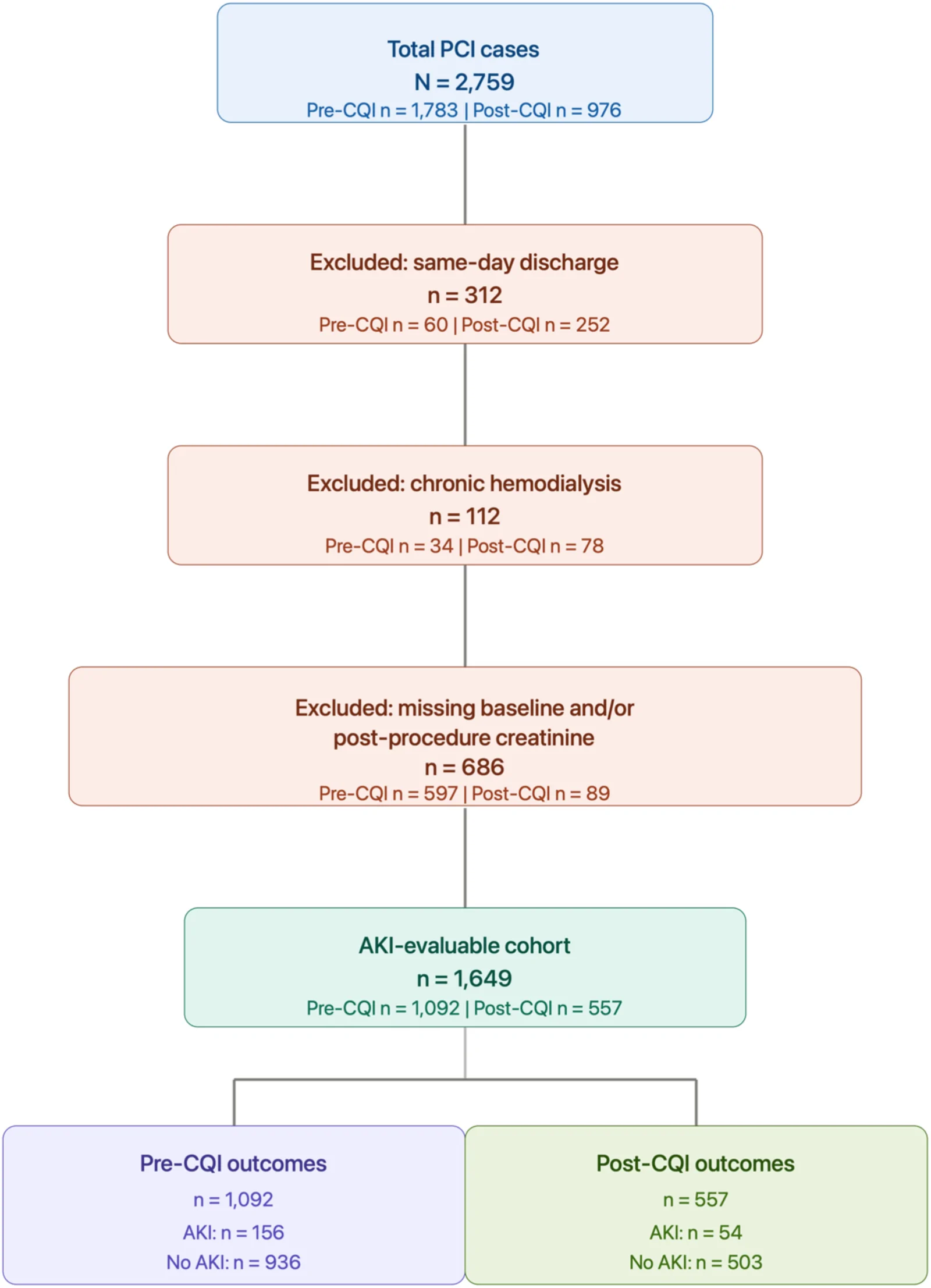
**Flow diagram of patients included in AKI analysis.** Of 2759 PCI cases, patients were excluded from AKI analysis due to same-day discharge (n = 312; pre-CQI n = 60, post-CQI n = 252), chronic hemodialysis (n = 112; pre-CQI n = 34, post-CQI n = 78), or missing baseline and/or postprocedure creatinine data after these exclusions (n = 686; pre-CQI n = 597, post-CQI n = 89). The final AKI-evaluable cohort included 1649 cases, including 1092 pre-CQI cases and 557 post-CQI cases. AKI occurred in 156 pre-CQI cases and 54 post-CQI cases. AKI, acute kidney injury; CQI, continuous quality improvement; PCI, percutaneous coronary intervention.

### Bleeding events

Baseline and postprocedure hemoglobin values were available for all 2759 PCI cases, including patients discharged the same-day; therefore, no patients were excluded from the hemoglobin-drop analysis due to SDD status or missing hemoglobin data. The mean drop in Hgb per procedure also decreased from 0.65 to 0.54 g/dL (*P* = .004). Notably, blood transfusions were required more often post-CQI (4.0% vs 2.2% of cases, *P* < .001), whereas NCDR-defined observed bleeding did not differ between groups (2.0% vs 2.6%; *P* = .38). Mean preprocedure hemoglobin was lower in the post-CQI cohort (12.2 vs 12.7 g/dL, *P* = .01). The Holm-Bonferroni-adjusted *P* value for hemoglobin-drop was .008.

Other serious complications, including pericardial tamponade (0% vs 0.2%) and need for new dialysis (0.2% vs 0.6%), were rare in both groups (Table 2).

### SDD

SDD after PCI improved markedly; its rate increased from 13.0% to 24.7% post-CQI (*P* < .001). The increase in SDD was especially pronounced for elective outpatient cases and those finishing by early afternoon. Cases completed before 15:00 were far more likely to result in SDD in the post-CQI era than previously (for all cases done before 15:00, 22.5% SDD post-CQI vs 11.2% pre-CQI, *P* < .001; Table 3). The CQI committee instituted protocols to identify low-risk candidates for SDD (based on access site, hemostasis, ambulation readiness, and clinical stability) and to expedite their recovery. In contrast, cases finishing late in the day rarely achieved SDD due to logistic constraints (eg, postanesthesia care unit closing); this remained unchanged.

**Table 3:** Hospitalization course and case metrics

	Total	Pre-CQI (2018 - Oct 2022)	Post-CQI (Nov 2022 - 2024)	*P* value
Average case time, min	96.6 (139)	97.4 (154)	96.0 (110)	.77
Average LOS, d	3.5 (5.6)	3.3 (5.1)	3.8 (6.4)	.02
Without outliers^a^	2.1 (2.0)	2.1 (1.9)	2.1 (2.1)	.73
Average post-Cath LOS, d	1.8 (4.3)	1.6 (4.0)	2.1 (4.7)	<.001
Without outliers^a^	0.9 (1.2)	0.8 (1.2)	1.0 (1.3)	<.001
Cases done after 1500	780 (28.3)	503 (28.2)	277 (28.4)	.63
Cases done before 1500 + SDD^b^	419 (15.2)	199 (11.2)	220 (22.5)	<.001
Elective cases done before 1500 + SDD^b^	401 (14.5)	186 (10.4)	215 (22.0)	<.001
SDD^b^	472 (17.1)	231 (13.0)	241 (24.7)	<.001 (adj <.001)^c^
Discharge disposition				.48
Home	2550 (94.1)	1643 (93.6)	907 (94.9)	
Rehab/extended care	60 (2.2)	42 (2.4)	18 (1.9)	
Skilled nursing facility	54 (2.0)	38 (2.2)	16 (1.7)	
Other acute care hospital	35 (1.3)	26 (1.5)	9 (0.9)	
Against medical advice	7 (0.3)	4 (0.2)	3 (0.3)	
Other	5 (0.2)	2 (0.1)	3 (0.3)	

Values are mean ± SD or n (%). CQI, continuous quality improvement; SDD, same-day discharge; LOS, length of stay.

a 257 outliers with LOS > 8.5 days removed.

b SDD means being admitted and discharged the same-day as opposed to being discharged the day of Cath completion.

c Adjusted *P* values are Holm-Bonferroni corrected across the 3 prespecified primary end points (AKI, mean Hgb drop, and SDD). Adjusted *P* values are shown only for primary end points; all other *P* values are nominal/exploratory.

The average procedure duration did not change (96.0 vs 97.4 minutes, *P* = .77). An interrupted time-series analysis confirmed an upward trend in the monthly proportion of SDDs after CQI initiation (+0.7% per month, *P* = .004); there was no trend preintervention (Figure 2). The Holm-Bonferroni-adjusted *P* value for increase in SDD was <.001.

**Figure 2:**
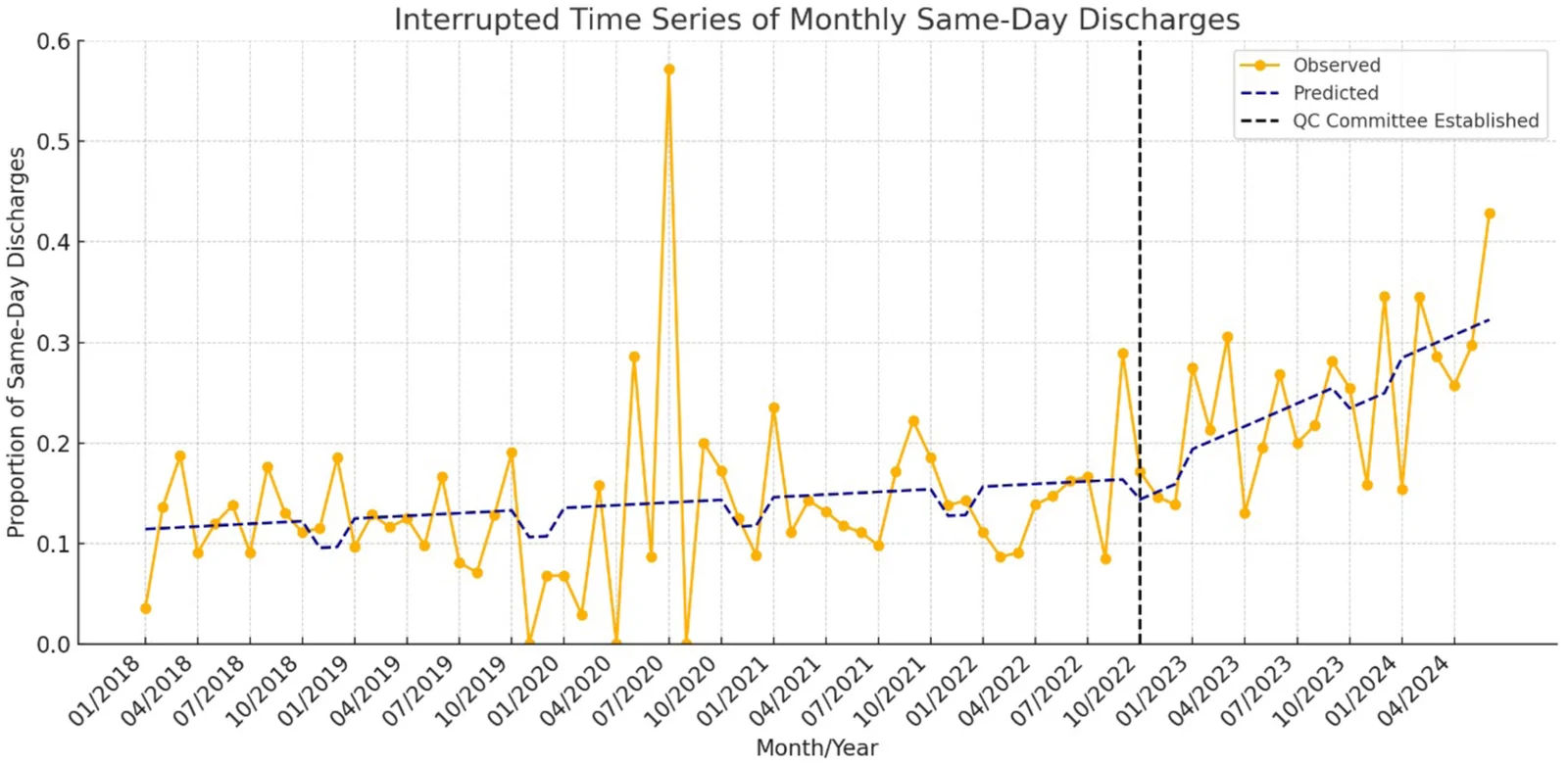
**Interrupted time-series of monthly same-day discharges.** Same-day discharge (SDD rates following PCI over time. (Left) Prequality committee (QC), the percentage of PCI patients discharged home the same-day was low with no upward trend (∼0.1% increase/mo, *P* = .13). (Right) Post-QC, the SDD rate increased steadily (∼0.7% per month, *P* = .004). By mid-2024, roughly 25% of PCI patients were discharged the same-day. PCI, percutaneous coronary intervention.

### Mortality

There were no differences in in-hospital mortality between the 2 periods (2.0% post vs 1.5% pre, *P* = .32), and the distribution of causes of death (eg, cardiac vs noncardiac) did not shift appreciably (Table 2).

### Disposition and length of stay

Despite higher patient acuity in the post-CQI cohort, most patients in both periods were discharged home (∼94% in each period) with only a small fraction requiring skilled nursing or rehab facility placement (Table 3). The overall post-PCI hospital mean length of stay (LOS) was slightly longer in the post-CQI group (2.1 days vs 1.6 days, *P* < .001). This increase in LOS likely reflects the higher complexity and need for monitoring in some post-CQI patients (eg, those with cardiogenic shock or requiring MCS).

### Process measures

#### PCI volume

Average monthly PCI volume increased from 30.7 cases per month in the pre-CQI period to 48.2 cases per month post-CQI (*P* < .001). Segmented regression analysis confirmed a steeper PCI volume increase post-CQI (+1.0 vs +0.2 cases/mo, *P* < .001 and *P* = .02, respectively) (Figure 3). These volume trends are presented as contextual markers of institutional throughput rather than as measures of quality. Along with increasing procedural volume, the clinical case mix evolved over time.

**Figure 3:**
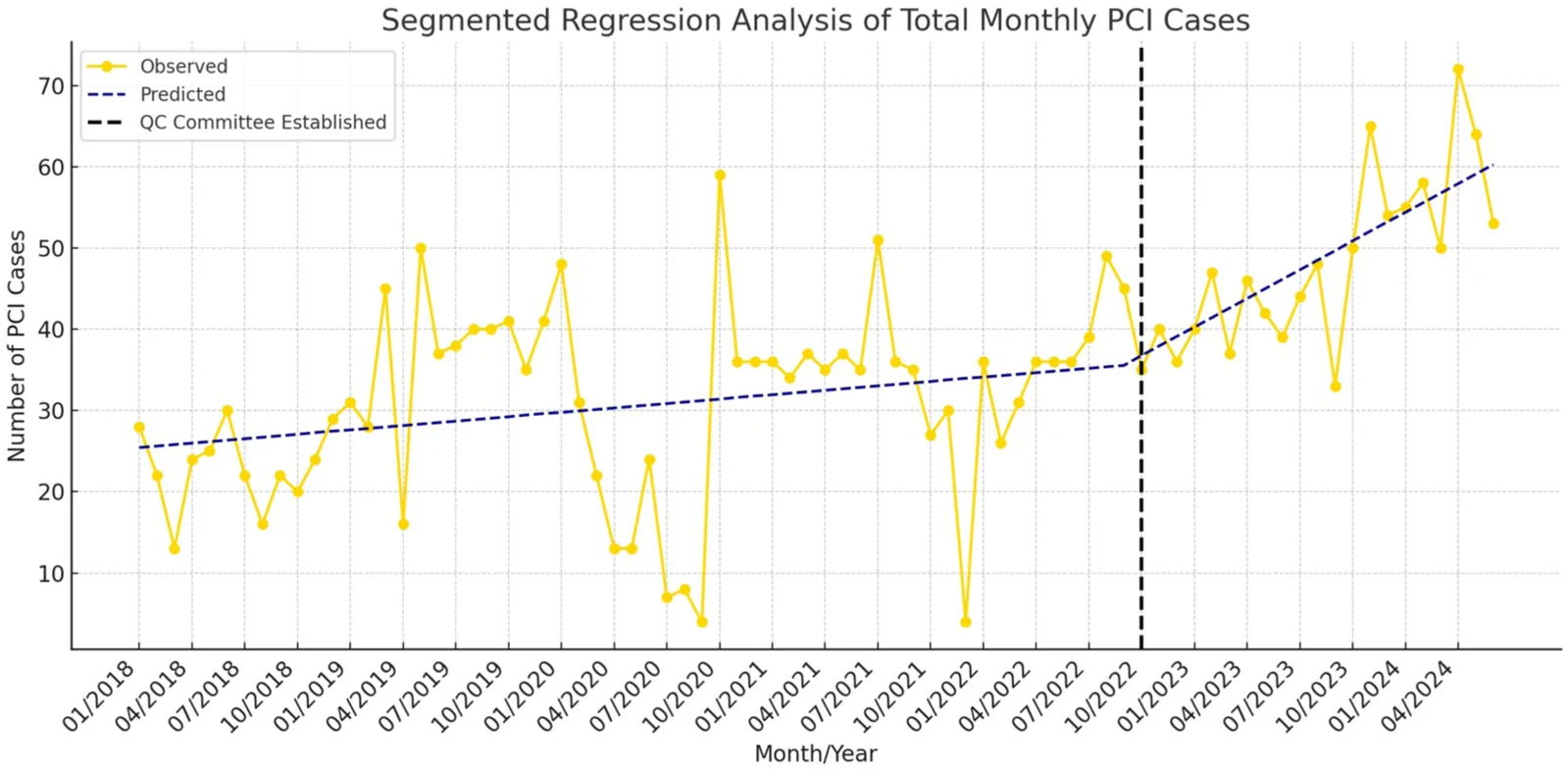
**Segmented regression analysis of total monthly PCI cases.** Monthly percutaneous coronary intervention (PCI) volume before and after quality committee (QC) implementation. (Left) pre-QC (2018 through October 2022), PCI volume increased gradually (slope ∼0.2 cases/month, *P* = .02). (Right) Post-QC (November 2022 onward), PCI volume increased more steeply (slope ∼1.2 cases/month, *P* < .001). An inflection in the volume trend is evident after CQI initiation (vertical dashed line). CQI, continuous quality improvement.

#### Radial access use rate

After CQI implementation, there were changes in procedural practice patterns. Radial arterial access use increased (33.4% pre-CQI to 58.9% post-CQI, *P* < .001). This dramatic increase in radial access adoption is illustrated by an integrated time-series analysis, which showed an upward trend in the monthly proportion of radial cases after November 2022 (monthly increase of 1.2% post-CQI vs 0.2% pre-CQI, *P* < .001; Figure 4). The increased use of radial access aligns with one of the CQI committee’s key initiatives to reduce bleeding.

**Figure 4:**
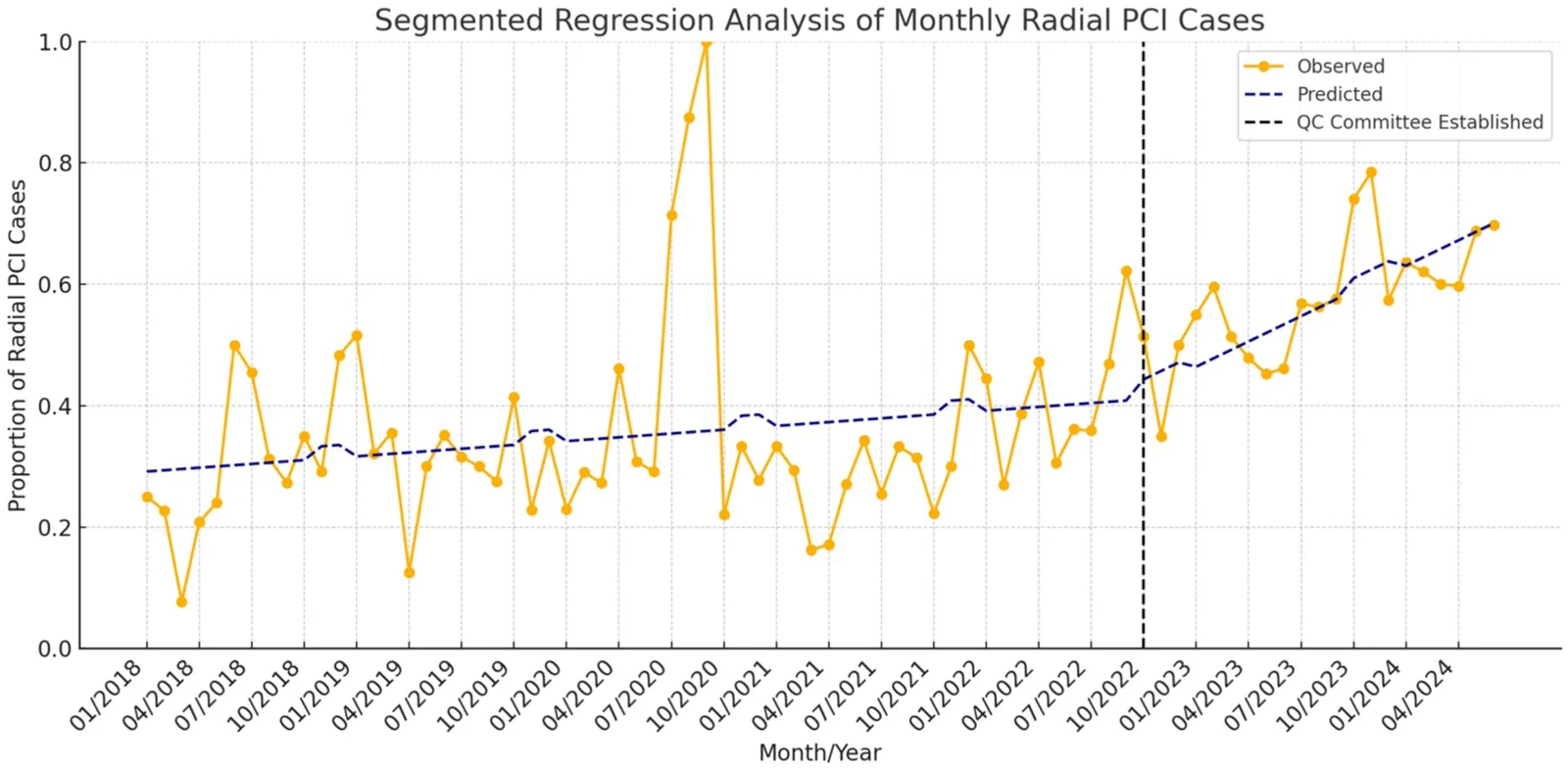
**Segmented regression analysis of monthly radial PCI cases.** Proportion of percutaneous coronary intervention (PCI) cases using radial access (vs femoral) over time. (Left) Prequality committee (QC), radial access use was slowly rising (∼0.2% increase per month, *P* = .049). (Right) Post-QC, radial access adoption accelerated (∼1.2% increase per month, *P* < .001).

#### Staged PCI

Concurrently, the incidence of multivessel staged PCI (planned second sessions) rose to 16.5% post-CQI from 3.1% pre-CQI (*P* < .001), consistent with greater procedural complexity and increased use of staged revascularization for complex coronary disease.

## Discussion

Despite higher procedural volume and patient complexity, CQI implementation was associated with improved PCI safety and discharge efficiency.

PCI volume rose substantially following CQI introduction, demonstrating improved institutional capacity and throughput. Although PCI volume is not included among current guideline-endorsed quality metrics, it is presented as a contextual indicator of procedural demand and institutional capacity, rather than as a direct measure of quality. The observed increase likely reflects evolving referral patterns and willingness to treat older and higher-risk patients, coinciding with the maturation of quality oversight processes.

Consistent with this shift, patients undergoing PCI in the post-CQI period were older and more likely to have heart failure, anemia, or severe frailty. Despite this higher-risk profile, several key outcome measures—including bleeding, AKI, in-hospital mortality, and same-day discharge—either improved or remained stable in the post-CQI period. These findings suggest that structured risk identification and mitigation strategies embedded within the CQI process may facilitate safe management of more complex patients. SDD rates nearly doubled post-CQI from 13% to 25%, whereas longer LOS among selected patients likely reflected appropriate monitoring of higher-risk cases.

The only outcome metric that worsened nominally was the intra- and postprocedural transfusion rate, which rose from 2.2% to 4.0% post-CQI, without an increase in bleeding events, likely due to treating more patients with baseline anemia and increased usage of MCS, without a corresponding increase in NCDR-defined observed bleeding (2.0% vs 2.6%, *P* = .38; Table 2). The CQI committee identified pre-PCI anemia optimization and blood-conservation strategies as future CQI targets.^20^^,^^21^

One of the most impactful changes was increasing radial access adoption from one-third to nearly 60% of cases. Radial access is well-known to reduce access site bleeding and has become standard of care, as evidenced by data here showing no retroperitoneal bleeds post-CQI.^20^^,^^21^ By minimizing IV contrast (via CQI-driven alerts), usage dropped, reducing AKI incidence. These findings underscore how targeted protocols improve outcomes.

Overall, after the CQI committee was introduced, process measures improved substantially, and key outcome measures either improved or remained stable despite a marked increase in procedural volume and the treatment of older, more comorbid, and frail patients. The principal committee-led interventions and associated changes in PCI outcomes are summarized in the Central Illustration.

**Central Illustration:**
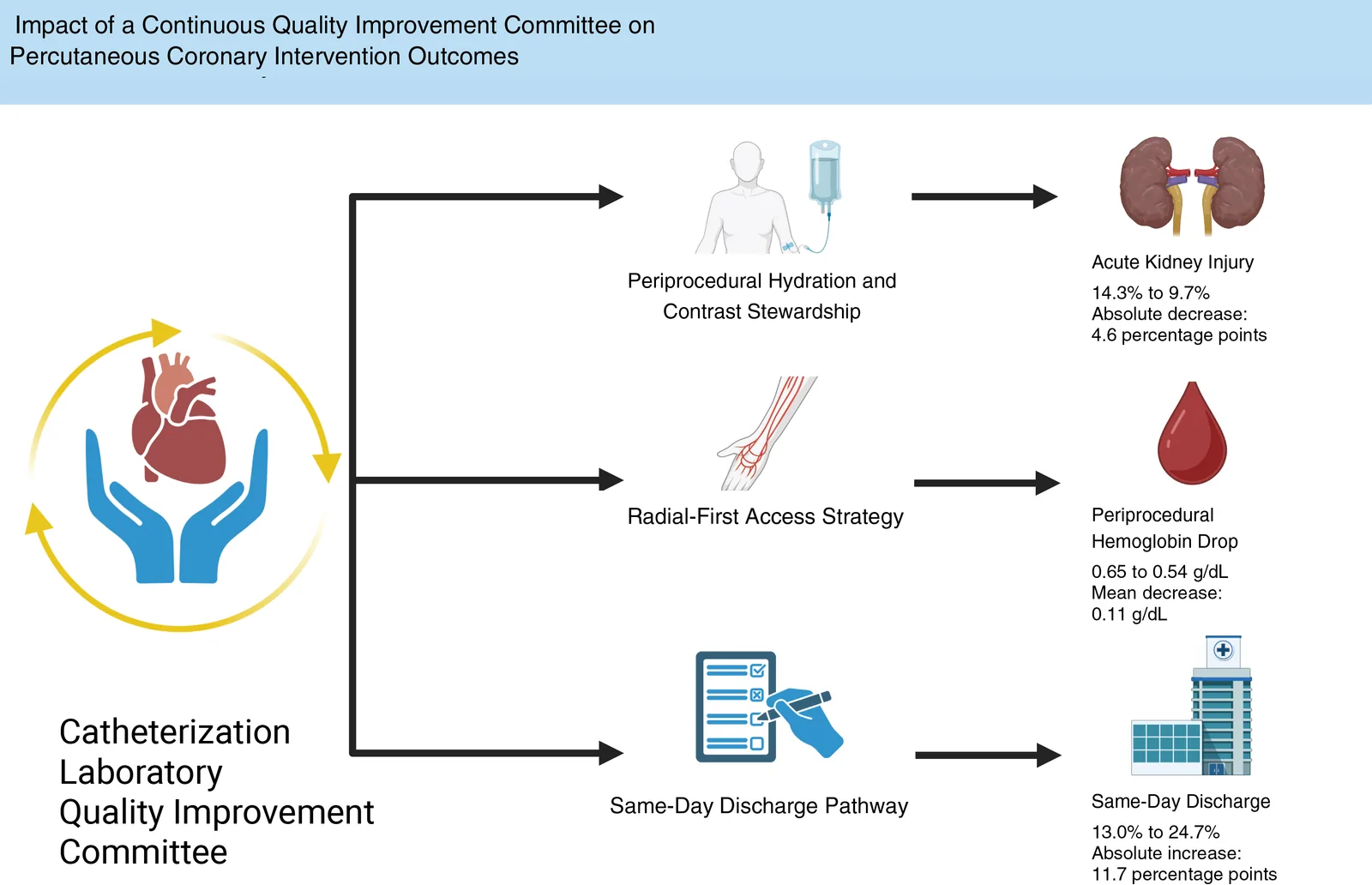
**Impact of a continuous quality improvement committee on percutaneous coronary intervention outcomes.** Implementation of a multidisciplinary catheterization laboratory CQI committee introduced standardized contrast stewardship and hydration protocols, a radial-first access strategy, and a structured same-day discharge pathway. Following implementation, acute kidney injury decreased from 14.3% to 9.7%, mean periprocedural hemoglobin-drop decreased from 0.65 to 0.54 g/dL, and same-day discharge increased from 13.0% to 24.7%. CQI, continuous quality improvement; PCI, percutaneous coronary intervention. Created with BioRender.com.

### COVID-19 implications

The COVID-19 pandemic overlapped with the study period and may have affected PCI volumes and case acuity, which should be considered when interpreting temporal trends.^22^^,^^23^

### Limitations

Several limitations of this study should be acknowledged. First, as an observational retrospective study, causality cannot conclusively be attributed to the CQI program for the improvements observed. For example, recent NCDR data demonstrates a trend toward lower contrast utilization during PCI.^24^ Multiple converging factors, including evolving guideline recommendations, increasing awareness of contrast-associated kidney injury risk, and adoption of contrast sparing approaches such as radial access and intravascular imaging, have driven national trends toward contrast minimization.^25^ Although these trends may have contributed to improvements in renal outcomes and complicate isolation of the program’s independent effect, they are unlikely to fully account for the magnitude of renal outcome improvement observed following CQI implementation. Second, the data were derived from registry entries and electronic medical records, which may lack granular details on procedural technique or operator decision-making nuances. Some improvements might be related to factors not captured (for example, introduction of a new device or a change in personnel). Third, this is a single-center experience in an academic urban hospital, and results may not generalize to all settings. Finally, the higher-risk profile of post-CQI patients indicates a selection bias: by design, the committee encouraged PCI in patients who may have been prior noncandidates at this center. Although this expands care, it complicates direct pre-/postoutcome comparisons because the patient populations differed. Both risk adjusted and unadjusted outcomes are reported where possible to provide a balanced view.

AKI analyses were restricted to cases with evaluable baseline and postprocedure creatinine and excluded patients on chronic dialysis and those discharged the same-day, consistent with CathPCI Registry AKI definitions and the NCDR AKI risk model methodology.^12^ Incomplete creatinine capture may differ by clinical workflow and discharge practices—particularly with increased same-day discharge—which could introduce selection bias and limit comparability between periods. Exclusion counts and AKI findings are reported in the context of incomplete creatinine data.^26^, ^27^, ^28^

Additionally, postdischarge outcomes among SDD patients were not systematically captured during the study period. Although SDD eligibility required clinical stability, successful ambulation, access site hemostasis, absence of periprocedural complications, reliable transportation, and a follow-up plan, standardized postdischarge phone call data, emergency department visits, readmissions, and postdischarge mortality were not available for analysis. Therefore, these SDD findings should be interpreted as reflecting successful SDD implementation and in-hospital discharge readiness rather than comprehensive postdischarge safety.

## Conclusions

CQI committee implementation was associated with improved PCI safety and efficiency, including lower AKI, fewer access complications, and higher SDD rates despite higher-risk case mix. These findings support targeted CQI programs as a practical strategy to improve care delivery for high-risk PCI patients.

## CRediT authorship contribution statement

**Arnav Kumar Singla:** Writing – review & editing, Writing – original draft, Visualization, Project administration, Investigation, Formal analysis, Data curation, Conceptualization. **Basmah Fayaz:** Writing – review & editing, Methodology, Investigation, Conceptualization. **Christopher Heinrich:** Writing – review & editing, Visualization, Methodology. **Samuel Miller:** Writing – original draft, Conceptualization. **John Simpson:** Writing – original draft, Investigation, Conceptualization. **Samantha Mutai:** Writing – review & editing, Writing – original draft, Resources, Methodology. **Gilgamish Maloul:** Writing – review & editing, Writing – original draft, Resources, Investigation. **Malek Lababidi:** Writing – review & editing, Writing – original draft, Resources, Investigation. **Julie Ahrenholz:** Visualization, Project administration, Methodology, Formal analysis, Data curation, Conceptualization. **Weili Zheng:** Writing – review & editing, Writing – original draft, Supervision, Methodology. **Faisal Hasan:** Writing – review & editing, Writing – original draft, Supervision, Project administration, Investigation. **Hussam S. Suradi:** Writing – review & editing, Supervision, Project administration, Conceptualization.

## Declaration of competing interest

The authors declared no potential conflicts of interest with respect to the research, authorship, and/or publication of this article.
