# Supplementary material for: Impact of a Continuous Quality Improvement Committee on Outcomes for Patients Undergoing Percutaneous Coronary Intervention: A Single-Center Retrospective Cohort Study
Source: J Soc Cardiovasc Angiogr Interv. 2026 Aug 20;5(10):105577. doi: 10.1016/j.jscai.2026.105577 (PMC13700054; doi:10.1016/j.jscai.2026.105577)
Supplement: Supplementary file 1 [file mmc1.docx]

**Appendices**

**Appendix A** Cath CQI methods and protocol

This appendix outlines the protocols implemented to optimize postprocedure care including minimizing vascular complications, prevention of contrast-induced AKI, and ensure medication reconciliation. These protocols are embedded in an electronic order set through the electronic medical record system which contains “hard stop” checkpoints requiring physician input to ensure all relevant orders are appropriately signed prior to the patient leaving the catheterization laboratory.

## 1. Access site care and hemostasis protocols

### Femoral access protocol

- **Bleeding avoidance strategies:** Routine limited iliofemoral angiography is performed in all cases to evaluate access site anatomy and determine is closure device use is feasible. Vascular closure devices (VCDs; eg, Angio-Seal or Perclose) are utilized whenever anatomically feasible.
- **Bedrest and activity orders (mandatory hard stop):** Flat bedrest duration is determined by hemostasis method and procedure type and is subject to extension at physician discretion based on access complexity or heparin dosing.

| **Hemostasis method** | **Diagnostic procedure** | **Interventional procedure (PCI)** |
| --- | --- | --- |
| **Manual compression** | 3 hours strict bedrest | 5 hours strict bedrest |
| **Vascular closure device (VCD)** | 2 hours strict bedrest | 4 hours strict bedrest |

- **Intra-aortic balloon pump (IABP) management:** Bedrest is ordered indefinitely until device removal.
  - Maintain an assist frequency of 1:1.
  - Assess peripheral pulses (pedal and brachial) every 1 hour for the first 24 hours, then every 2 hours and as needed (PRN).
  - Strict prohibition of blood draws from the balloon pump line.
- **Hematoma/bleeding nursing communication:** Automatically checked order instructing nursing staff to monitor the puncture site for drainage, discoloration, or hematoma. In the event of active bleeding or hematoma expansion: apply manual pressure 1 to 2 cm superior to the puncture site for 10 minutes, apply a pressure dressing once hemostasis is achieved, and immediately notify the cardiology service.

### Radial access protocol

- **Peripheral vascular assessment:** Performed matching the postprocedure vital sign schedule. Documentation requires evaluation of pulse strength, sensation, capillary refill, skin temperature, and skin color.
  - *Upper extremity:* Must include wrist flexion and extension strength alongside hand sensation.
  - *Lower extremity (if applicable):* Must include ankle dorsiflexion and plantarflexion strength alongside foot sensation.
- **TR band hemostasis management:** Initial decompression timing is stratified by total intraprocedural heparin administration.

Heparin ≤5000 units───>Wait 1 hour───>Remove 3 mL air Q15M until TR band empty

Heparin >5000 units───>Wait 2 hours──>Remove 3 mL air Q15M until TR band empty

**Bleeding decompression rule:** If bleeding or hematoma occurs during air withdrawal, re-inject the exact volume of air removed to restore hemostasis. Maintain pressure for 30 minutes before attempting further decompression. Once the TR Band is completely removed, observe the site for 15 minutes prior to discharge. For complex cases with prolonged procedural times or elevated activated clotting times (ACT), the initial hold time may be extended to 3 or 4 hours at physician discretion.

- **Minor radial oozing:** If minor bleeding occurs from the port site, inject 2 mL of air into the compression device port (not to exceed 18 mL total system volume) and notify the cardiology fellow.

## 2. Post-cath hydration protocol (CI-AKI mitigation)

This order set represents a mandatory hard stop for all diagnostic and interventional coronary cases to prevent contrast-induced nephropathy.

- **Inclusion criteria:** Baseline renal insufficiency defined as an estimated glomerular filtration rate (eGFR) less than 60 mL/min/1.73 m², or any documented baseline renal dysfunction in complex procedural settings.
- **Exclusion criteria:** Acute decompensated heart failure, clinical pulmonary edema, severe valvular heart disease, chronic hemodialysis, or end-stage renal disease (ESRD).
- **Maximum safe contrast threshold:** Calculated using the following standardized formula: maximum contrast volume (mL) = 3.7 × eGFR.^19^
- **Left ventricular end-diastolic pressure (LVEDP)-guided hydration:** Fluid administration rates are time-limited to 4 hours postprocedure based on invasive hemodynamic measurements or baseline systolic function as outlined below:

| Hemodynamic/functional criteria | Intravenous fluid rate (intra- and postprocedure) | Duration |
| --- | --- | --- |
| LVEDP <13 mm Hg | 300 mL/h | 4 h |
| LVEDP 13-18 mm Hg | 3 mL/kg/h | 4 h |
| LVEDP >18 mm Hg (or if no LVEDP obtained) | 1.5 mL/kg/h | 4 h |
| No risk factors/excluded | IV fluids not ordered via this protocol | — |

## 3. Discharge and quality metrics hard stops

The electronic order set prevents case closure and discharge summary generation until the following elements are completed:

- **Dual antiplatelet therapy (DAPT):** Mandatory order reconciliation for post-PCI antiplatelet agents (aspirin and P2Y12 inhibitors) ensuring appropriate dosing and duration. Orders are also sent to the patient’s listed pharmacy to ensure uninterrupted DAPT administration.
- **Cardiac rehabilitation referral:** An automated hard stop requires either ordering or deferring cardiac rehabilitation for all cases. Eligibility criteria for cardiac rehabilitation is outlined in the order to ensure that eligible post-PCI or acute coronary syndrome (ACS) patients receive prompt referral.
- **Patient education material:** If a closure device is employed, an order is automatically selected to include device-specific patient education materials (tailored to the specific closure device or radial compression band used) in the patient’s discharge summary.

**Appendix B** Baseline and procedural characteristics among patients included in the AKI analysis.

|  | Overall AKI-evaluable (n = 1649) | Pre-CQI (n = 1092) | Post-CQI (n = 557) | *P* value |
| --- | --- | --- | --- | --- |
| Average age (y) (SD) | 66.0 (12.7) | 65.0 (12.0) | 68.0 (13.0) | <.001 |
| Race, n (%) |  |  |  | .09 |
| White | 968 (58.7) | 630 (57.7) | 338 (60.7) |  |
| Black | 543 (32.9) | 370 (33.9) | 173 (31.1) |  |
| Asian | 76 (4.6) | 47 (4.3) | 29 (5.2) |  |
| Hispanic/Latino | 279 (16.9) | 174 (15.9) | 105 (18.9) |  |
| Other | 23 (1.4) | 20 (1.8) | 3 (0.5) |  |
| Sex, n (%) |  |  |  | .22 |
| Male | 1049 (63.6) | 706 (64.7) | 343 (61.6) |  |
| Female | 600 (36.4) | 386 (35.3) | 214 (38.4) |  |
| Avg BMI (SD) | 30.0 (7.0) | 30.0 (7.0) | 30 (6.90) | .24 |
| Medical history, n (%) |  |  |  |  |
| CVA | 274 (16.6) | 183 (16.8) | 91 (16.3) | .83 |
| PAD | 185 (11.2) | 128 (11.7) | 57 (10.2) | .37 |
| Chronic lung disease | 174 (10.6) | 114 (10.4) | 60 (10.8) | .84 |
| Prior PCI | 561 (34.0) | 364 (33.3) | 197 (35.4) | .41 |
| Diabetes | 799 (48.5) | 537 (49.2) | 262 (47.0) | .41 |
| Cardiogenic shock | 65 (3.9) | 37 (3.4) | 28 (5.0) | .11 |
| Anemia | 268 (16.3) | 173 (15.8) | 95 (17.1) | .53 |
| Heart failure | 501 (30.4) | 291 (26.6) | 210 (37.7) | <.001 |
| MCS needs, n (%) | 123 (7.5) | 82 (7.5) | 41 (7.4) | .99 |
| Preprocedure Cr, median (IQR) | 1.1 (.87-1.5) | 1.1 (.88-1.5) | 1.1 (.86-1.4) | .03 |
| Contrast volume (mL) (SD) | 177 (80.0) | 189 (78.0) | 153 (78.8) | <.001 |
| eGFR, median (IQR) | 70.3 (47.2-87.8) | 71.2 (46.8-88.2) | 69.2 (47.8-86.7) | .90 |
| CKD stage, n (%) |  |  |  |  |
| No CKD | 1023 (62.0) | 682 (62.5) | 341 (61.2) | .63 |
| I-II | 236 (14.3) | 149 (13.6) | 87 (15.6) | .28 |
| III | 161 (9.8) | 85 (7.8) | 76 (13.6) | <.001 |
| IV-V | 229 (13.9) | 176 (16.1) | 53 (9.5) | <.001 |
| Access, n (%) |  |  |  | <.001 |
| Radial | 660 (40.0) | 341 (31.2) | 319 (57.1) |  |
| Femoral | 989 (60.0) | 751 (68.8) | 238 (42.7) |  |

Same-day discharge patients, chronic hemodialysis patients, and patients missing baseline and/or postprocedure creatinine were excluded from the AKI-evaluable cohort. Continuous variables are presented as mean ± SD or median (IQR), as appropriate. Categorical variables are presented as n (%). *P* values for median (IQR) variables were calculated using the Wilcoxon rank-sum test.

AKI, acute kidney injury; CQI, continuous quality improvement; BMI, body mass index; CVA, cerebrovascular accident; PAD, peripheral arterial disease; PCI, percutaneous coronary intervention; MCS, mechanical circulatory support; eGFR, estimated glomerular filtration rate; CKD, chronic kidney disease; Cr, creatinine

**Appendix C** Multivariable logistic regression for acute kidney injury among the AKI-evaluable cohort.

|  | Adjusted OR | 95% CI | *P* value |
| --- | --- | --- | --- |
| Post-CQI period | 0.63 | 0.45-0.87 | .006 |
| Age, per year | 1.00 | 0.99-1.02 | .55 |
| Heart failure | 1.02 | 0.73-1.43 | .92 |
| Baseline eGFR | 1.00 | 1.00-1.01 | .067 |
| Cardiogenic shock | 2.07 | .96-4.48 | .064 |
| MCS | 0.78 | 0.41-1.50 | .46 |

Model adjusted for age, heart failure, baseline eGFR, cardiogenic shock, and mechanical circulatory support.

OR, odds ratio; CI, confidence interval; CQI, continuous quality improvement; eGFR, estimated glomerular filtration rate; MCS, mechanical circulatory support.
